# Virus Latency and the Impact on Plants

**DOI:** 10.3389/fmicb.2019.02764

**Published:** 2019-12-06

**Authors:** Hideki Takahashi, Toshiyuki Fukuhara, Haruki Kitazawa, Richard Kormelink

**Affiliations:** ^1^Laboratory of Plant Pathology, Graduate School of Agricultural Science, Tohoku University, Sendai, Japan; ^2^Plant Immunology Unit, International Education and Research Center for Food Agricultural Immunology, Graduate School of Agricultural Science, Tohoku University, Sendai, Japan; ^3^Department of Applied Biological Sciences and Institute of Global Innovation Research, Tokyo University of Agriculture and Technology, Tokyo, Japan; ^4^Food and Feed Immunology Group, Laboratory of Animal Products Chemistry, Graduate School of Agricultural Science, Tohoku University, Sendai, Japan; ^5^Livestock Immunology Unit, International Education and Research Center for Food Agricultural Immunology, Graduate School of Agricultural Science, Tohoku University, Sendai, Japan; ^6^Laboratory of Virology, Wageningen University, Wageningen, Netherlands

**Keywords:** beneficial interactions with plant viruses, endogenous viral elements, latent infection, stress tolerance, plant virus

## Abstract

Plant viruses are thought to be essentially harmful to the lives of their cultivated crop hosts. In most cases studied, the interaction between viruses and cultivated crop plants negatively affects host morphology and physiology, thereby resulting in disease. Native wild/non-cultivated plants are often latently infected with viruses without any clear symptoms. Although seemingly non-harmful, these viruses pose a threat to cultivated crops because they can be transmitted by vectors and cause disease. Reports are accumulating on infections with latent plant viruses that do not cause disease but rather seem to be beneficial to the lives of wild host plants. In a few cases, viral latency involves the integration of full-length genome copies into the host genome that, in response to environmental stress or during certain developmental stages of host plants, can become activated to generate and replicate episomal copies, a transition from latency to reactivation and causation of disease development. The interaction between viruses and host plants may also lead to the integration of partial-length segments of viral DNA genomes or copy DNA of viral RNA genome sequences into the host genome. Transcripts derived from such integrated viral elements (EVEs) may be beneficial to host plants, for example, by conferring levels of virus resistance and/or causing persistence/latency of viral infections. Studies on viral latency in wild host plants might help us to understand and elucidate the underlying mechanisms of latency and provide insights into the *raison d’être* for viruses in the lives of plants.

## Introduction

So far, virologists have focused only on a parasitic relationship between plant viruses and their host plants. In most described cases, the interaction between viruses and host plants negatively affects host morphology and physiology, resulting in disease (Hull, 2014). In a majority of cases, viruses are virulent and cause disease in crops during their mono-cultivation in open fields or greenhouses for food production. Not surprisingly, the current taxonomy of plant viruses is primarily based on viruses isolated from cultivated crops showing disease symptoms (Wren et al., 2006; Owens et al., 2012). However, a survey on latent infections of plant hosts has revealed that some plants may be infected with viruses without any clear symptoms (Roossinck, 2005, 2010; Richert-Pöggeler and Minarovits, 2014). More recent studies using a metagenomic approach have revealed that asymptomatic infections of plants with viruses might be a much more common event in nature than initially thought (Kreuze et al., 2009; Barba et al., 2014; Stobbe and Roossinck, 2014; Kamitani et al., 2016; Pooggin, 2018; Zhang et al., 2018). Asymptomatic infections may result from tolerance, in which plants do not suffer from wild type (high titer) virus replication levels, or from viral persistence, in which virus titers are reduced to avoid cytopathic effects and harm to the host. During virus latency, no viral replication occurs, and viruses remain in a kind of silenced/dormant status. Since a number of definitions of tolerance to viruses exist in genetics, physiology, and ecology, the relation of tolerance to virus titer still remains to be discussed (Little et al., 2010; Råberg, 2014). In the following sections, an overview will be given of plant virus latency. During this entire overview, discussion of viral latency also refers to viral persistence, since many studies have not distinguished between latency and persistence. Possible underlying mechanisms will be discussed, as well as how plant virus latency/persistence may be harmful or beneficial to the life of plants.

## Plant Viruses That Can Perform Latent Infection

**FIGURE 1:**
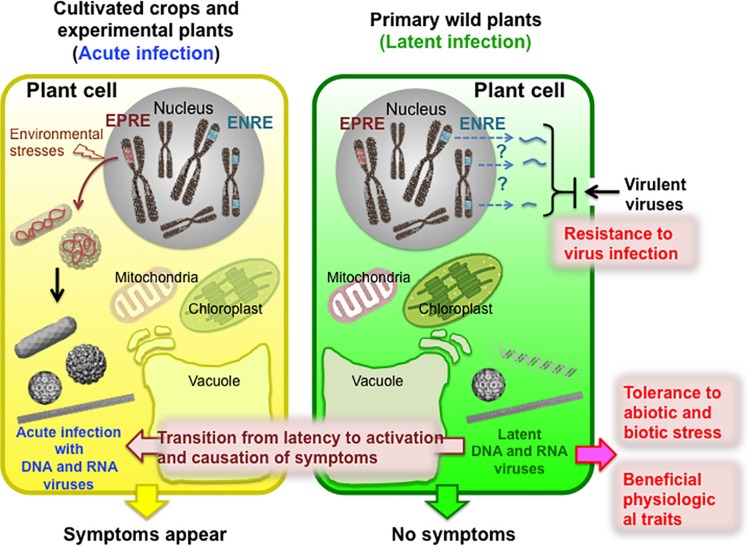
Schematic of positive and negative effects of latent virus infection on wild host plants in nature. Wild plants are often latently/persistently infected with viruses without exhibiting any apparent symptoms. Moreover, endogenous viral elements, such as endogenous pararetroviral elements (EPREs) and endogenous non-retroviral elements (ENREs), have become integrated into the genomes of some host plant species. The latent infection can occasionally transition to activation into an acute infection accompanied by the appearance of disease symptoms. This activation can occur due to vector-mediated transmission to cultivated crops, mixed infections with other viruses, environmental stresses, or the particular developmental stage of plant growth. However, latent/persistent infections can be of benefit to plants, such as by conferring resistance to infection by another virus, tolerance to abiotic or biotic stresses, or beneficial physiological traits that improve the lives of host plants.

Wild plants are often latently infected with viruses in nature without any apparent disease symptoms (Min et al., 2012; Shates et al., 2019). Table 1 is a list of plant viruses that are reported to latently infect primary wild host plants and to transition from latency to activation in crops or experimental host plants with the appearance of disease symptoms. Latent viruses are apparently easily maintained in perennial plants (Hull, 2014). In the case of annual plants, latent viruses may be transmitted to the next generation of host plants through pollination, although the efficiency of seed-borne transmission depends on the virus and host plant (Hull, 2014). However, viruses that latently infect wild plants often cause disease symptoms in closely related crop plants through vector-mediated transmission and in experimental plants *via* mechanical wounding (Table 1) (Pagán et al., 2012; Roossinck and Garcia-Arenal, 2015). Moreover, in perennial plants asymptomatically infected with viruses, a transition from latency to activation and the appearance of disease symptoms occasionally occurs during mixed infections with other viruses, changing environmental conditions, or during certain host plant growth stages (Table 1 and Figure 1).

**TABLE 1:** List of plant viruses featuring a latent infection and possible transition from the latency to the causation of symptoms^(1)^.

**Virus genome**	**Virus name**	**Taxonomy**		**Virus particle**	**Virus genome structure**	**Genomic segmentation**	**Primary host plants/Biological life cycle**	**Transmission**	**Transition from latency to activation and causation of symptoms/Crops or experimental host plants**	**References**
									
		**Family**	**Genus**							
**ssRNA**										
	*Apple latent spherical virus* (ALSV)	*Comoviridae*	Cheravirus	Icosahedral	ss(+)RNA	Bipartite	*Malus pumila/*Perrenial	Seed	Vein clearing, chlorotic spots and distortion/*Chenopodium quinoa*	Koganezawa et al., 1985; Li et al., 2000
	*Apricot latent virus* (ApLV)	*Betaflexiviridae*	Foveavirus	Filamentous	ss(+)RNA	Monopartite	*Prunus armeniaca/* Perennial	Grafting, mechanical	Yellow asteroid or sooty ring spots/*Prunus* species	Nemchinov and Hadidi, 1998; Nemchinov et al., 2000; Grimová and Ryšánek, 2012
	*Grapevine algerian latent virus* (GALV)	*Tombusviridae*	Tombusvirus	Icosahedral	ss(+)RNA	Monopartite	Algerian grapevine (*Vitis* spp.)/ Perennial	Soil, mechanical	Chlorotic or necrotic spots along the veins of the leaves/Grapevine cultivars	Gallitelli et al., 1989; Lovato et al., 2014
	*Heracleum latent virus* (HLV)	*Betaflexiviridae*	Vitivirus	Filamentous	ss(+)RNA	Monopartite	*Heracleum sphondylium/* Perennial	Aphid, mechanical	Mottle/*Amaranthaceae*; chlorosis/*Umbelliferae*; vein-clearing/*Chenopodiaceae*	Bem and Murant, 1979
	*Lolium latent virus* (LoLV)	*Alphaflexiviridae*	Lolavirus	Filamentous	ss(+)RNA	Monopartite	*Lolium perenne*, *L. multiflorum*/ Perennial	Mechanical	Chlorotic or necrotic streaking on the leaves/*Lolium perenne* or *L. multiflorum*, or their hybrid	Huth et al., 1995; Maroon-Lango et al., 2006; Vaira et al., 2008
	*Plantago asiatica mosaic virus* (P1AMV)	*Alphaflexiviridae*	Potexvirus	Filamentous	ss(+)RNA	Monopartite	*Plantago asiatica*/Perennial	Mechanical	Severe necrosis/*Lilium* spp.	Kostin and Volkov, 1976; Solovyev et al., 1994; Ozeki et al., 2006
	*Olive latent virus 1* (OLV-1)	*Tombusviridae*	Necrovirus	Icosahedral	ss(+)RNA	Monopartite	*Olea europaea*/Perennial	Soil, mechanical	Occasional leaf chlorosis/Olive, Citrus and Tulip	Gallitelli and Savino, 1985; Félix et al., 2007
	*Poinsettia latent virus* (PnLV)	*Luteoviridae*	Polemovirus	Icosahedral	ss(+)RNA	Monopartite	*Euphorbia pulcherrima*/Perennial	Grafting	No symptom	Aus dem Siepen et al., 2005
	*Pothos latent virus* (PoLV)	*Tombusviridae*	Tombusvirus	Icosahedral	ss(+)RNA	Monopartite	*Epipremnum aureum*/Perennial	Soil	Mosaic and distortion of leaf blade/*Nicotiana benthamiana*	Rubino and Russo, 1997
	*Spinach latent virus* (SLV)	*Bromoviridae*	Ilarvirus	Icosahedral	ss(+)RNA	Bipartite	*Spinacia oleracea*/Annual	Seed, mechanical	Severe stunting/*Axyris amaranthoides*; Chlorotic vein banding, yellow mottling and growth reduction/*Chenopodium quinoa*	Bos et al., 1980
	*Spring beauty latent virus* (SBLV)	*Bromoviridae*	Bromovirus	Icosahedral	ss(+)RNA	Tripartite	*Claytonia virginica/*Perennial	Mechanical	Vein necrosis/*Nicotiana megalosiphon*; Mottle/*Commelina diffusa*; Systemic necrosis/*Gomphrena globosa*; Mottle/*Pisum sativum*	Valverde, 1985
	*Strawberry latent ringspot virus* (SLRSV)	*Secoviridae*	Cheravirus	Icosahedral	ss(+)RNA	Bipartite	*Fragaria* × *ananassa/* Perennial	Seed, nematodes	Chlorotic spots/*Anemone* × *hybrida*; Chlorotic streaks and necrotic rings/*Impatiens walleriana*; Vein chlorosis/*Tibouchina* sp.	Lister, 1964; Schmelzer, 1969; Tang et al., 2013; Mazyadr et al., 2014
**dsRNA**										
	Plant endornavirus^(2,3)^	*Endornaviridase*	Endornavirus	No true capsid	dsRNA	Monopartite	*Basellaceae, Cucurbitaceae, Fabaceae, Poaceae, Solanaceae, Aquifoliaceae*	Seed	No symptom	Roossinck, 2011a; Roossinck et al., 2011; Fukuhara, 2019
	*Southern tomato virus* (STV)	*Amalgaviridae*	Amalgavirus	No true capsid	dsRNA	Monopartite	*Solanum lycopersicum/*Annual	Seed	Discoloration and size reduction of the tomato fruits/*Solanum lycopersicum* cultivars	Sabanadzovic et al., 2009
	*White clover cryptic virus 1* (WCCV-1)	*Partitiviridae*	Alphacryptovirus	Icosahedral	dsRNA	Bipartite	*Trifolium repens*/Perennial	Seed	Suppression of root nodule formation when sufficient nitrogen is present/*Lotus japonicus*	Boccardo et al., 1985; Natsuaki et al., 1986; Nakatsukasa-Akune et al., 2005
	*White clover cryptic virus 2* (WCCV-2)	*Partitiviridae*	Betacryptovirus	Icosahedral	dsRNA	Bipartite	*Trifolium repens*/Perennial	Seed, pollen	No symptom	Natsuaki et al., 1986; Boccardo et al., 1987
	*Ryegrass cryptic virus* (RGCV)	*Partitiviridae*	Deltapartitivirus	Icosahedral	dsRNA	Bipartite	*Lolium perenne*/Perennial	Seed	No symptom	Guy and Sward, 1991
**dsDNA**										
	*Horseradish latent virus* (HRLV)	*Caulimoviridae*	Caulimovirus	Icosahedral	dsDNA	Monopartite	*Armoracia rusticana*/Perennial	Aphid	Mild chlorotic mottle with a faint yellow banding of the major vein of the leaves/*Brassica campestris*	Richins and Shepherd, 1986
**ssDNA**										
	*Euphorbia caput-medusae latent virus* (EcmLV)	*Geminiviridae*	Capulavirus	Icosahedral	ssDNA	Monopartite	*Euphorbia caput-medusae*/Perennial	Aphid	Leaf curling, distortion and yellowing/*N. benthamiana* and *S. lycopersicum*	Bernardo et al., 2013, 2016
	*Plantago lanceolata latent virus* (PlLV)	*Geminiviridae*	Capulavirus	Icosahedral	ssDNA	Monopartite	*Plantago lanceolata*/Perennial *Chenopodium quinoa*/Annual	Aphid	Not determined	Susi et al., 2017, 2019

*^(1)^Viruses that were primarily isolated from symptomatic cultivated plants and then discovered to latently infect wild plants without any symptoms were not included in this table. ^(2)^There is only one report that endornavirus-infected common bean (*Phaseolus vulgaris*) cv. Black Turtle Soup has beneficial physiological traits without visible pathogenic effects, as described in Table 3. ^(3)^Endornavirus has been characterized as dsRNA virus in Virus Taxonomy: Ninth Report of the International Committee on Taxonomy of Viruses (Owens et al., 2012). However, in Tenth ICTV Virus Taxonomy Profile (https://talk.ictvonline.org/ictv-reports/ictv_online_report/positive-sense-rna-viruses/w/endornaviridae), endornavirus consists of a single molecule of naked ss(+)RNA and its dsRNA is the viral replicative form, which is relatively stable and presents in relatively high quantities in the host tissue.*

### Latent Infection With RNA Viruses

Latent infections with positive single-stranded [ss(+)] RNA viruses are found in many host plants (Table 1). Viruses in those studies have primarily been isolated from asymptomatic cultivated plants or wild plants. However, latent infections occasionally convert into acute infections with the appearance of disease symptoms, as nicely observed in the following examples. Apricot latent virus (ApLV) was first isolated from asymptomatic *Prunus armeniaca* in Moldavia in 1993 (Zemtchik and Verderevskaya, 1993). However, some *Prunus* species, after being inoculated by grafting a healthy scion onto ApLV-infected *P. armeniaca* as a rootstock, become ApLV-infected and exhibit yellow asteroid or sooty ring spots on their leaves (Martelli and Jelkmann, 1998; Grimová and Ryšánek, 2012). Grapevine algerian latent virus (GALV) was first isolated in Italy from an Algerian vine (*Vitis* spp.) infected with grapevine fanleaf virus (GFLV) and is considered a latent virus due to the presence of only GFLV-related symptoms (Gallitelli et al., 1989). Passage of this mixed infection to nipplefruit (*Solanum mammosum*) and statice (*Limonium* spp.) causes severe stunting, chlorotic spots, and mosaic symptoms (Lovato et al., 2014). Heracleum latent virus (HLV) was isolated in Scotland from *Heracleum sphondylium* that showed no disease symptoms (Bem and Murant, 1979). When this virus is transmitted by aphids to host plants in the *Amaranthaceae*, *Chenopodiaceae*, and *Umbelliferae* families, it causes leaf mottle, chlorosis, and systemic vein-clearing symptoms (Bem and Murant, 1979). Plantago asiatica mosaic virus (PIAMV) was originally isolated from *Plantago asiatica* L. and latently infects a wide range of plant species, except cereals (Kostin and Volkov, 1976; Solovyev et al., 1994). Recently, PIAMV has been found in *Lilium* spp. (Oriental types) showing severe necrosis of the leaves (Ozeki et al., 2006). Knowledge on ss(+) RNA virus infections in native perennial grasses is slowly accumulating (Malmstrom and Alexander, 2016; Alexander et al., 2017) and also points to cases of viral latency. Lolium latent virus (LoLV) was initially detected in several areas in Europe, including Germany, the Netherlands, France, and the United Kingdom, as *Ryegrass latent virus* (Huth et al., 1995). LoLV has also been reported in the United States for the first time in ryegrass hybrids (*Lolium perenne* x *L. multiflorum*) (Maroon-Lango et al., 2006; Vaira et al., 2008). Plants infected with LoLV alone exhibit either no symptoms or mild chlorotic flecking, but the flecks coalesce to form chlorotic to necrotic streaking on the leaves depending on environmental conditions and growth stage.

Double-stranded (ds) RNA viruses from the families *Amalgaviridae*, *Chrysoviridae*, *Endornaviridae*, *Partitiviridae*, and *Totiviridae* are found in plants, fungi, and protists (Roossinck et al., 2011; Song et al., 2013). Other virus-like sequences related to those families have been discovered by *in silico* surveys using known dsRNA viral sequences as queries against the NCBI Expressed Sequence Tag (EST) database (Liu et al., 2012). Whereas plant endornaviruses and amalgaviruses do not form true particles, plant cryptoviruses of the family *Partitiviridae* form icosahedral particles (Table 1) (Owens et al., 2012). The latter viruses are usually present at low copy numbers, have no obvious effects on the host plant, and can be maintained as a latent infection during the life of a perennial or be efficiently transmitted vertically *via* gametes (Table 1) (Fukuhara, 2019). Southern tomato virus (STV) belongs to the amalgaviruses, which are also known to latently infect plants without forming true particles (Martin et al., 2011) and are thought to represent a transitional intermediate between totiviruses and partitiviruses (Sabanadzovic et al., 2009). However, a correlation between the presence of STV dsRNA and discoloration and size reduction of fruits in some tomato cultivars suggests that infection with STV may cause abnormal development depending on the cultivar (Sabanadzovic et al., 2009). White clover cryptic virus 1 (WCCV-1), WCCV-2, and ryegrass cryptic virus (RGCV) are members of the family *Partitiviridae*, that commonly appear in plants and fungi (Boccardo et al., 1985, 1987; Natsuaki et al., 1986; Guy and Sward, 1991). Interestingly, WCCV-1 is thought to play a role in the regulation of the host-rhizobium symbiosis (Nakatsukasa-Akune et al., 2005). WCCV-2 and RGCV do not induce clear symptoms on either their natural or experimental host plants (Table 1).

Although latent infections often correlate with the absence of symptoms, negative effects of such infection may be manifested in other plant traits and/or at certain developmental stages. In this sense, recent studies have indicated that asymptomatic infections may reduce plant survival depending on the growth stage (Fraile et al., 2017; Rodríguez-Nevado et al., 2017). However, despite these studies, the effect of virus infection on the survival of wild plants remains largely unknown.

### Latent Infection With DNA Viruses

The family *Caulimoviridae* includes all plant viruses with circular double-stranded DNA (dsDNA) genomes with a reverse transcription phase in their lifecycles. The caulimovirus Horseradish latent virus (HRLV), isolated from horseradish (*Armoracia rusticana*) (Table 1) (Richins and Shepherd, 1986), causes latent infections in the natural host plant *Armoracia rusticana*, but mild chlorotic mottling symptoms can be observed during infection of some *Brassica* plants with HRLV (Richins and Shepherd, 1986).

Members of the family *Geminiviridae* are characterized by a circular single-stranded DNA (ssDNA) genome that is encapsidated within a twinned icosahedral particle. Geminiviruses infect both monocotyledonous and dicotyledonous plants and cause major losses in agricultural production worldwide. Seven genera are recognized within this family, but, recently, two new additional genera, *Capulavirus* and *Grablovirus*, have been established (Varsani et al., 2017). Euphorbia caput-medusae latent virus (EcmLV) and plantago lanceolata latent virus (PILV), both classified as *Capulavirus*, latently infect their natural host plants, *Euphorbia caput-medusae* and *Plantago lanceolata*, respectively (Bernardo et al., 2016; Susi et al., 2017). PILV was discovered during a viral metagenomics survey of uncultivated *Plantago lanceolata* and *Chenopodium quinoa* plants that did not exhibit symptoms (117; 118). Symptom development has not been examined in crops or experimental plants challenged with PILV yet. Although EcmLV-inoculated *Euphorbia caput-medusae* do not exhibit any symptoms, EcmLV-inoculated *Nicotiana benthamiana* and tomato plants exhibit leaf curling, distortion, and yellowing (Bernardo et al., 2013).

From the growing number of reports described above, it becomes clear that wild host plants are often asymptomatically infected with viruses and that these may transform from latency to activation. Considering that the viruses reported and described above in relation to latency belong to different plant virus families with as many different lifestyles also raises the question of how these viruses end up in a stage of latency and whether or not there is an underlying generic mechanism to this.

Considering that latent virus infections might also improve the survival, genetic diversity, or population density of wild host plant species in nature, knowledge on their biology will provide insights that might lead to potential future exploitations for cultivated crops and be related to beneficial effects on plant hosts, e.g., increased resilience toward (a)biotic stress factors (see sections further below). Although the underlying mechanism of plant virus latency remains elusive, some cases implicate endogenized viral elements (EVEs) in this.

## Endogenous Viral Elements (EVEs): Cause of Viral Latency?

All types of viruses can become endogenous by the integration of (partial) viral (copy) DNA sequences into the genomes of various host organisms (Figure 1) (Holmes, 2011; Teycheney and Geering, 2011; Feschotte and Gilbert, 2012; Aiewsakun and Katzourakis, 2015). These sequences are often and generally referred to as endogenized viral elements (EVEs), the most well-known ones coming from animal/human-infecting retroviruses like human immunodeficiency virus (HIV) and several leukemia viruses. The replication of retroviral RNA genomes requires prior integration of a DNA copy of the entire viral RNA genome into the DNA of infected cells, mediated by a virus-encoded integrase. In a next step, transcription by the host machinery will produce progeny viral RNA. Thus, for retroviruses, endogenization is essential for the accomplishment of their life cycle, and these viruses are therefore debated as an example of EVEs. However, for none of the plant viruses known so far is the integration of a DNA copy required for their replication. During the past two decades, an increasing number of observations have been made on integrated plant viral sequences in the genome of various plant species. Most of them involve the integration of a partial viral genome sequence, but a few cases have been reported on the endogenization of entire viral genome sequences.

The first EVEs to be described in plants contained sequences that originated from two groups of plant viruses, both containing circular DNA genomes, i.e., the single-stranded DNA geminiviruses and the double-stranded DNA pararetroviruses (reviewed by Harper et al., 2002; Hohn et al., 2008; Iskra-Caruana et al., 2010). Meanwhile, EVEs have been found originating from a diverse group of plant nuclear and cytoplasmic replicating DNA and RNA viruses, respectively (Table 2) (Owens et al., 2012). Furthermore, integrated sequences have been reported that originate from ancestral viruses (Chiba et al., 2011; Chu et al., 2014; Diop et al., 2018). Nowadays, EVEs are commonly distinguished into two groups: those originating from pararetroviral elements (Endogenous pararetroviral elements [EPREs]) (Diop et al., 2018) and those containing any other plant virus sequence (Endogenous non-retroviral elements [ENREs]) (Chiba et al., 2011; Chu et al., 2014). Since many EPREs and ENREs integrated a long time ago, they are suggested to represent ancient relics of viral infection, and their study is called Paleovirology. However, recent studies indicate that these might not just represent molecular fossils but could play a role in pathogenicity or contribute to levels of resistance (Bertsch et al., 2009). Considering the latter, EVEs may well play a major role in the establishment of viral latency/persistence.

**TABLE 2:** List of viruses occuring integration viral genome into nuclear genome and mitcondria DNA.

**Type of endogenous viral element (EVE)**	**Integrated form in host chromorome**	**Episomal virus**	**Endogenous viral elements or their constructed virus**	**Ancestral virus family**	**Ancestral virus genus**	**Ancestral virus genome structure**	**Primary host plants**	**Impact on host life^(1)^**	**References**
**Endogenous pararetroviral element (EPRE)**									
	Full-length	Banana streak virus (BSV)		Caulimoviridae	Badnavirus	dsDNA	*Musa acuminata*, *M. balbisiana*	Activatable EPREs integrated into plant genome; Transition from latency to the causation of symptoms *via* episomes	Harper et al., 1999; Ndowora et al., 1999
	Full-length	Dahlia mosaic virus (DMV)		Caulimoviridae	Caulimovirus	dsDNA	Dahlia variabilis	Activatable EPREs integrated into plant genome; Transition from latency to the causation of symptoms *via* episomes	Eid and Pappu, 2014
	Full-length	Petunia vein clearing virus (PVCV)		Caulimoviridae	Petuvirus	dsDNA	*Petunia hybrida*	Activatable EPREs integrated into plant genome; Transition from latency to the causation of symptoms *via* episomes	Harper et al., 2002; Richert-Pöggeler et al., 2003
	Full-length	Tobacco vein clearing virus (TVCV)		Caulimoviridae	Cavemovirus	dsDNA	*Nicotiana edwardsonii*	Activatable EPREs integrated into plant genome; Transition from latency to the causation of symptoms *via* episomes	Jakowitsch et al., 1999; Lockhart et al., 2000
	Segmented		Orendovirus^(1)^	Caulimoviridae		dsDNA	*Oryza sativa*	Non-activatable EPREs integrated into plant genome	Geering et al., 2010; Kunii et al., 2004
	Segmented		Florendovirus	Caulimoviridae		dsDNA	21 species	Non-activatable EPREs integrated into plant genome	Geering et al., 2014
	Segmented		Solendovirus	Caulimoviridae		dsDNA	3 species	Non-activatable EPREs integrated into plant genome	Jakowitsch et al., 1999; Matzke et al., 2004; Staginnus et al., 2007
	Segmented		Tomato EPRVs (LycEPRVs)	Caulimoviridae		dsDNA	*Solanum lycopersicum* and *S. habrochaites*	Generation of siRNAs from LycEPRV	Staginnus et al., 2007
**Endogenous non-retoroviral element (ENRE)**									
	Segmented		Partitivirus	Partitiviridae		dsRNA	10 monocot and 19 eudicot species	Non-activatable ENREs integrated into plant genome	Chiba et al., 2011; Liu et al., 2010; Chu et al., 2014
	Segmented		Endornavirus	Endornaviridae		dsRNA^(2)^	1 species	Non-activatable ENREs integrated into plant genome	Chu et al., 2014
	Segmented		Chrysovirus	Chrysoviridae		dsRNA	3 species	Non-activatable ENREs integrated into plant genome	Chu et al., 2014
	Segmented		Totivirus	Totiviridae		dsRNA	1 species	Non-activatable ENREs integrated into plant genome	Chu et al., 2014
	Segmented		Amalgavirus	Amalgaviridae		dsRNA	3 species	Non-activatable ENREs integrated into plant genome	Liu et al., 2010
	Segmented		Cytorhabdovirus	Rhabdoviridae		ss(−)RNA	9 plant families	Non-activatable ENREs integrated into plant genome	Chiba et al., 2011
	Segmented		Varicosavirus	Rhabdoviridae		ss(−)RNA	9 plant families	Non-activatable ENREs integrated into plant genome	Chiba et al., 2011
	Segmented		Potyvirus	Potyviridae		ss(+)RNA		Non-activatable ENREs integrated into plant genome	Tanne and Sela, 2005
	Segmented		Cucumovirus	Bromoviridae		ss(+)RNA		Non-activatable ENREs integrated into plant genome	Chiba et al., 2011
	Segmented		Geminivirus	Geminiviridae		ssDNA		Non-activatable ENREs integrated into plant genome	Bejarano et al., 1996; Ashby et al., 1997; Murad et al., 2004; Liu et al., 2011

*^(1)^Rice tungro bacilliform virus (RTBV) has been identified as an infectious agent of rice tungro diseases, independently of its endogenous pararetoroviral elements. Thus, endogenous RTBV-like sequence (eRTBVL) is an alias for segmented Orendovirus. ^(2)^Endornavirus has been characterized as dsRNA virus in Virus Taxonomy: Ninth Report of the International Committee on Taxonomy of Viruses (Owens et al., 2012). However, in Tenth ICTV Virus Taxonomy Profile (https://talk.ictvonline.org/ictv-reports/ictv_online_report/positive-sense-rna-viruses/w/endornaviridae), endornavirus consists of a single molecule of naked ss(+)RNA and its dsRNA is the viral replicative form, which is relatively stable and presents in relatively high quantities in the host tissue.*

### Endogenous Pararetroviral Elements

Most plant EPREs that have been characterized are derived from viruses in the family *Caulimoviridae*. The *Caulimoviridae* currently consists of eight genera: *Badnavirus*, *Caulimovirus*, *Cavemovirus*, *Petuvirus*, *Rosadnavirus*, *Solendovirus*, *Soymovirus*, and *Tungrovirus*, and the two tentative genera *Orendovirus* and *Florendovirus* (Table 2) (Bousalem et al., 2008; Geering et al., 2010). EPRV-like sequences derived from banana streak virus (BSV) in *Musa* spp., dahlia mosaic virus (DMV) in *Dahlia* spp., petunia vein-clearing virus (PVCV) in *Petunia* spp., tobacco vein-clearing virus (TVCV) in *Nicotiana* spp., and rice tungro bacilliform virus (RTBV) in *Oryza* spp. have been identified in their host genomes (Table 2) (Harper et al., 1999; Jakowitsch et al., 1999; Ndowora et al., 1999; Lockhart et al., 2000; Richert-Pöggeler et al., 2003; Eid and Pappu, 2014). EPREs corresponding to entire viral DNA genomes can generate episomal infections from their endogenous intact sequences within the host genome of specific cultivars in response to stress (Staginnus and Richert-Pöggeler, 2006). One of the most beautiful cases reports on an enemy from within a banana hybrid containing an endogenized copy of the Banana streak virus (BSV) genome (Iskra-Caruana et al., 2010). Episomal forms of BSV, DMV, TVCV, and PVCV apparently transition from latency to activation with the assembly of virus particles and symptoms of virus infection (Table 2) (Harper et al., 2002). Episomal copies may also be generated by transcription from tandemly arranged integrants or recombination from fragmented integrants in host genomes (Ndowora et al., 1999; Richert-Pöggeler et al., 2003). Interspecific crosses and *in vitro* propagation can induce EPRE reactivation, which has been shown to be economically detrimental in banana breeding (Chabannes and Iskra-Caruana, 2013).

In contrast to the relatively small number of reports on EPREs in which a full-length genome-copy has been integrated, most EPREs are non-infective because their sequences have been fragmented by deletions, mutations, or epigenetic modifications in plant genomes (Staginnus and Richert-Pöggeler, 2006; Staginnus et al., 2009). Segments of rice tungro bacilliform virus (RTBV) DNA have been identified between AT-dinucleotide repeats within several loci in rice genome databases (Kunii et al., 2004; Liu et al., 2012; Chen and Kishima, 2016). Furthermore, partial endogenous RTBV seems to have been generated by transcription from tandemly arranged integrants of RTBV or by recombination from fragmented integrants of RTBV in rice genomes (Chen et al., 2014). While active intact endogenous RTBV DNA has not been obtained from the rice genome, RTBV has been identified as the infectious agent of rice tungro diseases independently of its EPREs. Similarly, analysis of genomic sequences of *Solanum lycopersicum* and *S. habrochaites* revealed sequence similarity between their EPREs, named *Lyc*EPRVs, interspersed in these tomato genomes, indicating that they are potentially derived from one pararetrovirus (Staginnus et al., 2007). Furthermore, TA simple sequence repeats from endogenous florendoviruses have extensively colonized the genomes of two monocotyledonous plant species and 19 dicotyledonous plant species (Geering et al., 2014).

### Endogenous Non-retroviral Elements

Endogenous non-retroviral elements (ENREs) in the genomes of host plants are derived from segmented and rearranged viral sequences of dsRNA, ssDNA, or ssRNA viruses (Table 2) (Bejarano et al., 1996; Ashby et al., 1997; Murad et al., 2004; Tanne and Sela, 2005; Liu et al., 2010; Chiba et al., 2011; Liu et al., 2012; Chu et al., 2014). ENREs in host plants predominantly match RNA-dependent RNA polymerase (RdRP)-like, movement protein (MP)-like, and coat protein (CP)-like sequences of dsRNA viruses (Liu et al., 2010, 2012; Chiba et al., 2011; Chu et al., 2014). The genomes of various host plants have also been observed to contain sequences from negative-ssRNA [ss(-)RNA] viruses (Chiba et al., 2011). These sequences are homologous to CP-like sequences of cytorhabdovirus and varicosavirus. The integration of sequences homologous to positive-ssRNA [ss(+)RNA] in host plant genomes has also been demonstrated (Tanne and Sela, 2005; Chiba et al., 2011). Nucleotide sequences homologous to a part of the CP gene and 3′-UTR of a potyvirus or to a portion of the CP and movement protein (MP) genes of cucumber mosaic virus (CMV) have been identified in the genomic databases of grape and *Medicago truncatula*, respectively. Many ENREs derived from these RNA viruses appear to be long interspersed elements created by reverse transcriptase and integrase activities encoded by host nuclear genomes or by non-homologous recombination between viral RNA and RNA generated from a retrotransposon.

Repetitive geminivirus-related DNA (GRD) sequences have also been discovered in host genomes and appear to have resulted from promiscuous integration of multiple repeats of the geminivirus initiation (Rep) sequence into the nuclear genome of an ancestor of some host plant species (Bejarano et al., 1996; Ashby et al., 1997; Murad et al., 2004; Liu et al., 2011).

## Beneficial Effects of Virus Infection and EVEs on Host Plants

In general, few studies have focused on the benefits of virus latent infection and EVEs for host plants or the mutualistic symbioses between these and their host organisms (Figure 1). Viruses with beneficial functions for or mutualistic symbioses with various host organisms, including bacteria, insects, fungi, and animals, have been discovered and are being given more attention relatively recently (Barton et al., 2007; Nuss, 2008; Roossinck, 2011b). For example, many pathogenic bacteria produce a broad range of virulence factors that have turned out not to be expressed from the bacterial genome but rather from a phage genome (Brüssow et al., 2004; Boyd, 2012). Many wasps deposit symbiogenic polydnavirus during egg deposition in a lepidopteran caterpillar host to expresses “wasp” genes that suppress host immune responses and prevent encapsulation of the egg, allowing the larva to develop and mature normally (Edson et al., 1981).

A mutualistic three-way symbiosis involving a fungal virus, curvularia thermal tolerance virus (CThTV), the fungal endophyte C*urvularia protuberate*, and the panic grass *Dichanthelium lanuginosum* helps the grass to tolerate high temperatures and grow in geothermal soils (Redman et al., 2002; Márquez et al., 2007; Roossinck, 2015a, b). CThTV infection induces the expression of genes involved in the synthesis of trehalose and melanin, which confer abiotic stress tolerance in the fungal endophyte and the plant (Morsy et al., 2010). While, slowly, the idea of mutualistic and beneficial effects of viruses on their host has become generally accepted, the effects may differ (mechanistically) between those caused by acute viral infections, viral latency, and/or EVEs.

### Beneficial Effects of Acute Infection on Host Plant Lives

While, a decade ago, Roossinck and colleagues discovered and emphasized the beneficial effects of viral infections for host plants (Xu et al., 2008; Roossinck, 2011b), meanwhile, several virulent strains of plant viruses such as cucumber mosaic virus (CMV) Fny strain [CMV(Fny)], bromo mosaic virus (BMV) Russian strain, tobacco mosaic virus (TMV) U1 strain, and tobacco rattle virus (TRV) have been shown to confer drought or cold tolerance to their host plants (Table 3) (Xu et al., 2008; Roossinck, 2013; Westwood et al., 2013). Although the molecular mechanisms underlying this conferred drought and cold tolerance have not yet been elucidated, several metabolites, including osmoprotectants and antioxidants that are associated with improved drought and cold tolerance, were observed to increase in these virus-infected plants (Xu et al., 2008).

**TABLE 3:** List of viruses that infect host plants with beneficial effects on host plant lives.

**Virus name**	**Taxonomy**		**Virus particle**	**Virus genome**	**Genomic segmentation**	**Primary host plants/biological life cycle**	**Experimental host plants**	**Impact on the lives of crop or experimental plants**	**References**
								
	**Family**	**Genus**							
**Acute viruses**									
*Bromo mosaic virus* (BMV) strain Russian	*Bromoviridae*	Bromovirus	Icosahedral	ss(+)RNA	Tripartite		*Oryza sativa, Nicotiana benthamiana*	Drought tolerance	Xu et al., 2008
*Cucumber mosaic virus* (CMV) strain Fny	*Bromoviridae*	Cucumovirus	Icosahedral	ss(+)RNA	Tripartite		*Beta vulgaris, N. benthamiana, Chenopodium amaranthicolor, Arabidopsis thaliana*	Drought and cold tolerance, alteration of pollinator preference, tolerance to deterioration	Xu et al., 2008; Mauck et al., 2010; Roossinck, 2011b; Westwood et al., 2013; Groen et al., 2016; Bueso et al., 2017
*Tobacco mosaic virus* (TMV) strain U1	*Tobamoviridae*	Tobamovirus	Rod-shaped	ss(+)RNA	Monopartite		*Nicotiana tabacum, N. benthamiana*	Drought tolerance	Xu et al., 2008
*Tobacco rattle virus* (TRV)	*Virgaviridae*	Tobravirus	Rod-shaped	ss(+)RNA	Bipartite		*N. benthamiana*	Drought tolerance	Xu et al., 2008
*White clover mosaic virus* (WClMV)	*Alphaflexiviridae*	Potexvirus	Rod-shaped	ss(+)RNA	Monopartite		*Trifolium repens*	Less attractive to fungus gnats	van Molken et al., 2012
*Zucchini yellow moaic virus* (ZYMV)	*Potyviridae*	Potyvirus	Flexious	ss(+)RNA	Monopartite		*Cucurbita pepo*	Reducing plant susceptibility to powdery mildew infection	Harth et al., 2018
*Zucchini yellow moaic virus* (ZYMV)	*Potyviridae*	Potyvirus	Flexious	ss(+)RNA	Monopartite		*Cucumis sativa*	Less attractive to cucumber beetle, a vector of *Erwinia tracheiphila*	Shapiro et al., 2013
**Latent viruses**									
*Beet cryptic virus* (BCV) 1 and 2	*Partitiviridae*	Alphacryptovirus Deltapartitivirus	Icosahedral	dsRNA	Bipartite	*Beta vulgaris*/ Biennial	*Beta vulgaris*	Prevent yield losses under drought in persistently infected plants	Xie et al., 1994
*Cucumber mosaic virus* (CMV) strain Ho	*Bromoviridae*	Cucumovirus	Icosahedral	ss(+)RNA	Tripartite	*Arabidopsis halleri*/Perennial	*Arabidopsis thaliana*	Heat and drought tolerance, promotion of main root growth but suppression of lateral root development	Takahashi et al. in review
*Pepper cryptic virus 1* (PCV-1)	*Partitiviridae*	Deltapartitivirus	Icosahedral	dsRNA	Bipartite	*Capsicum annuum*/ Annual	*Capsicum annuum*	Manipulation of aphid behavior, which is a vector of acute viruses	Safari et al., 2019
*Phaseolus vulgaris endornavirus* (PvEV)	*Endornaviridase*	Endornavirus	No true capsid	dsRNA^(1)^	Monopartite	*Phaseolus vulgaris*/Annual	*Phaseolus vulgaris*	Beneficial physiological traits without visible pathogenic effect	Khankhum and Valverde, 2018
*Southern tomato virus* (STV)	*Amalgaviridae*	Amalgavirus	No true capsid	dsRNA	Monopartite	*Solanum lycopersicum/*Annual	*Solanum lycopersicum*	increased plant height, production of more fruit, higher germination rate of seeds in cultivar M82^(2)^	Fukuhara et al., 2019
*White clover cryptic virus 1* (WCCV-1)	*Partitiviridae*	Alphacryptovirus	Icosahedral	dsRNA	Bipartite	*Trifolium repens/Perennial*	*Trifolium repens*	Suppression of root nodule formation when sufficient nitrogen is present	Nakatsukasa-Akune et al., 2005

*^(1)^Endornavirus has been characterized as dsRNA virus in Virus Taxonomy: Ninth Report of the International Committee on Taxonomy of Viruses (Owens et al., 2012). However, in Tenth ICTV Virus Taxonomy Profile (https://talk.ictvonline.org/ictv-reports/ictv_online_report/positive-sense-rna-viruses/w/endornaviridae), endornavirus consists of a single molecule of naked ss(+)RNA and its dsRNA is the viral replicative form, which is relatively stable and presents in relatively high quantities in the host tissue. ^(2)^The phenotypes given by infection with STV were only observed in *Solanum lycopersicum* cultivar M82.*

In another recent study, the emission profile of volatile organic compounds from CMV(Fny)-infected *Solanum lycopersicum* and *Arabidopsis thaliana* altered the foraging behavior of bumblebees (*Bombus terrestris*), thereby increasing buzz pollination (Table 3) (Groen et al., 2016). Although CMV(Fny) infection decreased seed yield without buzz-pollination, the increased buzz-pollination in CMV(Fny)-infected plants raised their seed yields to levels comparable to those in mock-inoculated plants (Groen et al., 2016). Virus infections thereby positively affect plant reproduction through increased pollinator preference. Furthermore, *A. thaliana* plants infected with CMV(Fny) rendered seeds with improved tolerance to deterioration when compared to the non-inoculated plants (Table 3) (Bueso et al., 2017).

Plant viruses may also influence the susceptibility/preference of plants to biotic stressors of different natures. White clover mosaic virus (WCIMV) infection in *Trifolium repens* can decrease the attractiveness of white clover plants for female fungus gnats (van Molken et al., 2012). In zucchini yellow mosaic virus (ZYMV)-infected *Cucumis sativa*, attraction of the cucumber beetle, which can transmit the bacterial wilt pathogen *Erwinia tracheiphila*, was reduced (Shapiro et al., 2013). In both cases, the production of volatile compounds altered due to virus infection and appeared to protect host plants by decreasing herbivore infestation rates (van Molken et al., 2012; Shapiro et al., 2013). Thus, viruses clearly may have beneficial functions for plants in plant–herbivore interactions that could either involve attraction or repelling of certain insects. The effects of viral infections on plants are not limited to herbivores but are also reported in relation to fungal infections of plants. A comparative study using ZYMV-inoculated and non-inoculated controls cultivated in a greenhouse revealed that ZYMV-infected plants were more resistant to powdery mildew than the controls and that this seemed to be caused by elevated concentrations of salicylic acid, leading to enhanced pathogen defense responses (Harth et al., 2018).

### Beneficial Effects of Latent Infection on Host Plant Lives

Infection with most of the well-characterized viruses in crop plants is often acute; however, several plant species can be latently infected with viruses without showing symptoms (Roossinck, 2010, 2012a,b). Studies on viruses that latently infect host plants are still quite limited, and likewise the beneficial effects of latent virus infection, but this is slowly receiving growing interest (Table 3). Meanwhile, the protection of some plant virus strains that cause latent or mild infections against other more virulent strains has been well reported for many combinations of viruses and host plants so far (e.g., Agüero et al., 2018). For example, beet cryptic virus (BCV), a member of the family *Partitiviridae*, can prevent yield losses caused by drought conditions in latently infected *Beta vulgaris* (Xu et al., 2008). In *Lotus japonicus*, the artificial over-expression of the white clover cryptic partitivirus-1 (WCCV-1) coat protein (CP) gene, which is also a member of the family *Partitiviridae*, inhibited root nodule formation (Nakatsukasa-Akune et al., 2005). Although one could debate on the beneficial effects, these findings could indicate, albeit speculatively, that a latent infection with WCCV-1 suppresses excessive root nodule formation that otherwise would disrupt the growth of plants. *Phaseolus vulgaris* endornavirus (PvEV)-infected common bean (*Phaseolus vulgaris*) cv. Black Turtle Soup exhibits obvious beneficial physiological traits without visible pathogenic effects (Table 3) (Khankhum and Valverde, 2018). PvEV-infected plants exhibit faster seed germination, a longer radicle and pods, higher carotene content, and higher 100-seed weight (Khankhum and Valverde, 2018). Recently, cucumber mosaic virus (CMV) strain Ho (CMV[Ho]) has been isolated from asymptomatic *Arabidopsis halleri*, a perennial wild plant (Takahashi et al. in review). *A. halleri* is latently infected with CMV(Ho), and the virus has spread systemically. When this strain was inoculated on *A. thaliana*, the plants were persistently infected with CMV(Ho). CMV(Ho)-infected plants exhibited heat and drought tolerance and had promoted main root growth but suppressed lateral root development (Table 3). Furthermore, latent infection of *Solanum lycopersicum* cultivar M82 with southern tomato virus (STV) increased the plant height, production of fruit, and germination rate of seeds (Fukuhara et al., 2019). In another recent study (Safari et al., 2019), latent infection of *Capsicum annuum* by the partitivirus pepper cryptic virus 1 (PVC-1) interestingly appeared to deter aphids. This contrasted with studies in which acute infections with cucumber mosaic virus (CMV) attracted aphids to promote and facilitate the spread of CMV. The relation between PVC-1 and pepper thus appears to be beneficial, as it may protect the host from aphids transmitting acute viruses and aphid herbivory.

Altogether, it has become clear that virus infections, irrespective of whether acute or latent, can provide beneficial effects to host plants. While the underlying mechanisms leading to those beneficial effects have not yet been widely studied, it is not unlikely that these may be similar for acute and latent/persistent infections.

### Beneficial Effects of Endogenous Viral Elements on Host Plant Lives

A growing number of studies indicate the widespread nature of EVEs, including their presence in the genomes of various plant species (Holmes, 2011; Feschotte and Gilbert, 2012; Aiewsakun and Katzourakis, 2015). Although EVEs only lead to a viral infection in a few cases in plants, their presence has also been linked to beneficial effects. A study by Staginnus et al. (2007) demonstrated that cultivated tomato (*Solanum lycopersicum*) and a wild relative (*S. habrochaites*) both contained tomato EVEs with high sequence similarity, likely derived from the same pararetrovirus (*Lyc*EPRV). While the pathogenicity of *Lyc*EPRVs could not be demonstrated, transcripts derived from multiple *Lyc*EPRV loci and short RNAs complementary to *Lyc*EPRVs were detected in healthy plants and became elevated in abundance upon infection with heterologous viruses encoding suppressors of post-transcriptional gene silencing (Staginnus et al., 2007). This observation supports the idea that transcriptionally expressed EVEs may contribute to antiviral defense responses.

Although EVEs are thought to represent viral relics from the past resulting from a horizontal gene transfer (HGT) event following a viral infection, studies, as described above, indicate that EVEs can be responsive and may contribute to pathogenicity or virus resistance in the host (Bertsch et al., 2009). Whether viral integration into host genomes is ultimately of net benefit or harm to the host remains to be determined.

## Conclusion and Outlook

To date, most studies of plant viruses have focused on acute infection of cultivated crop plants. Many wild (non-cultivated) host plants are often asymptomatically/latently infected with viruses, and it is tempting to assume that this is the result of a natural evolution with benefits for the virus in terms of survival and dissemination and for the plant hosts in terms of survival and resilience to (a)biotic stressors.

Understanding the biology of latent infections is not only of scientific interest; the knowledge generated might also contribute to future exploitation in cultivated crops and be related to beneficial effects, e.g., increased resilience to (a)biotic stress factors.

Whether latent virus infections result from (partial) genome integration of viral sequences into the host genome, leading to EVEs, remains a matter of debate. However, support for the function of EVEs, and of the RNAi machinery, in the development of latent infections is being provided by recent studies in which persistence of RNA viruses in *Drosophila* was observed to result from reverse transcription and endogenization (or presence as episomal elements) of viral copy DNA (vDNA) sequences. Transcripts from those sequences were shown to be processed by the RNAi machinery, which inhibited viral replication (Goic et al., 2013). Application of reverse transcriptase inhibitors prevented the establishment of viral persistence, and, instead, lethal acute infections were observed. Following this study, more cases of the persistence of RNA viruses in insects were investigated and demonstrated to involve reverse transcription into vDNA (Goic et al., 2016; Nag et al., 2016). Latent infections, but also integrations of entire copies of a full-length viral genome, present a major risk toward cultivated crops due to the possibility of these viruses/EVEs becoming activated and giving rise to acute infections. A well-studied example of EVE-activation relates to endogenous BSV (eBSV) in hybrid banana (Iskra-Caruana et al., 2010). Interestingly, a recent study has successfully applied CRISPR/Cas9-mediated editing of the eBSV sequences and prevented proper transcription and activation into infectious particles (Tripathi et al., 2019). This not only paves the way to use hybrids containing dormant but activated infectious viral genome copies but also to use these inactivated hybrids in breeding programs to maximally exploit the potential beneficial effects of EVEs.

How can it be explained that cultivated agricultural crops seem to suffer more from viral disease symptoms, while their wild relatives/non-cultivated crops do not but seem more often to contain latent infections? Maybe this view is not correct, since plant virologists have so far been more interested in viruses causing disease and reducing crop yields and not those that do not cause harm (latent infections) or involve non-cultivated crops of no economic importance. On the other hand, if this view is correct, do agricultural crops suffer from viral disease more because they more often grown in monocultures that support the rapid spread of insect vector infestations into the entire outstanding crop, while non-cultivated crops are more resilient and protected due to a more natural balanced ecosystem? Or is there also an involvement of genetic traits, present in wild/non-cultivated crops but lost from cultivated crops during breeding for fast growth and high yields, that support the establishment of latent infections and result from millions of years of evolution? In this perspective, it is interesting to note that endornaviruses, which are normally found in persistent infections, have been observed in many different important crops but are only observed at a very low rate in wild plants. Although endornaviruses are not associated with visible pathogenic effects, their presence correlates with, e.g., faster seed germination, longer bean pods, higher seed weight, etc. (Fukuhara, 2019; Herschlag et al., 2019). Hence, it is very likely that endornaviruses have been positively selected for during breeding for economically important traits, but they rely on certain host factors to maintain their persistence and prevent viral disease. The absence of endornavirus from *Oryza* knocked down for certain components of the RNAi pathway (RDR or DCL) provided support for the involvement of the host cellular RNAi machinery in the maintenance of a persistent endornavirus infection (Urayama et al., 2010).

While many questions remain to be answered, e.g., the role of EVEs and/or RNAi in the establishment of persistence, studying latent infections in (non-cultivated) plants will be one of the challenges for the future (Watanabe et al., 2019). Further research is required not only to help understand this phenomenon but also to identify genetical traits that could keep viruses in a more dormant state while maintaining maximal benefits toward the host and suppressing negative effects from (a)biotic stressors.

## Author Contributions

HT and RK contributed substantially to the conception and design of this review article. HT, RK, TF, and HK co-wrote the manuscript. TF and HK reviewed the manuscript before submission for its intellectual content. All authors gave final approval of the published version.

## Conflict of Interest

The authors declare that the research was conducted in the absence of any commercial or financial relationships that could be construed as a potential conflict of interest.
